# Thermochemical hydrolysis of macroalgae *Ulva* for biorefinery: Taguchi robust design method

**DOI:** 10.1038/srep27761

**Published:** 2016-06-13

**Authors:** Rui Jiang, Yoav Linzon, Edward Vitkin, Zohar Yakhini, Alexandra Chudnovsky, Alexander Golberg

**Affiliations:** 1 The Porter School of Environmental Studies, Tel Aviv University, Tel Aviv, Israel; 2 Department of Mechanical Engineering, Tel Aviv University, Tel Aviv, Israel; 3 Department of Computer Science, Technion – Israel Institute of Technology, Haifa, Israel; 4 Department of Geography and Human Environment, Enviro-Digital Lab, Tel Aviv University, Israel

## Abstract

Understanding the impact of all process parameters on the efficiency of biomass hydrolysis and on the final yield of products is critical to biorefinery design. Using Taguchi orthogonal arrays experimental design and Partial Least Square Regression, we investigated the impact of change and the comparative significance of thermochemical process temperature, treatment time, %Acid and %Solid load on carbohydrates release from green macroalgae from *Ulva* genus, a promising biorefinery feedstock. The average density of hydrolysate was determined using a new microelectromechanical optical resonator mass sensor. In addition, using Flux Balance Analysis techniques, we compared the potential fermentation yields of these hydrolysate products using metabolic models of *Escherichia coli*, *Saccharomyces cerevisiae* wild type, *Saccharomyces cerevisiae* RN1016 with xylose isomerase and *Clostridium acetobutylicum*. We found that %Acid plays the most significant role and treatment time the least significant role in affecting the monosaccharaides released from *Ulva* biomass. We also found that within the tested range of parameters, hydrolysis with 121 °C, 30 min 2% Acid, 15% Solids could lead to the highest yields of conversion: 54.134–57.500 gr ethanol kg^−1^ *Ulva* dry weight by *S. cerevisiae* RN1016 with xylose isomerase. Our results support optimized marine algae utilization process design and will enable smart energy harvesting by thermochemical hydrolysis.

There is a pressing need for novel efficient and sustainable energy generation technologies. One of the pathways for energy conversion is to use bio-refineries, where biomass is converted into transportation fuels or other energy carriers. The proper choice of raw biomass material and of chemical and physical parameters are critical to ensuring the efficient production of sustainable food, feed, chemicals and biofuels. Current strategies for food production and renewable energy generation rely mostly on the classic land based agriculture. However, as indicated by the European Biorefinery Joint Strategic Research Roadmap for 2020: *A key issue for biomass production in Europe is land availability*^1^,^2^. Furthermore, concerns over net energy balance, land, potable water use, environmental hazards, and processing technologies question the relevance of cereal crops and lignocellulose biomass as sustainably addressing the near future food and energy challenges^3^. Difficulties in the cost-effectiveness of cultivation and dehydration currently prevent the implementation of broad scale microalgae technologies^4^. At the same time, an expanding body of evidence has demonstrated that marine macroalgae can provide a sustainable alternative source of biomass for sustainable food, fuel and chemical generation^5^,^6^. Marine sourced feedstocks, such as macroalgae (seaweed)^7^, have drawn researchers’ interest because they may overcome several negative environmental issues that characterize terrestrial crops, e.g., arable land, fertilizers and fresh water usage^8^,^9^.

In recent studies, we have formalized the thermodynamic constraints for the size and capacity of a single biorefinery^10^,^11^. The macroalgae from *Ulva* genus is of particular interest due to fast growth rates and high carbohydrate content^12^,^13^,^14^,^15^,^16^. We demonstrated this approach by a design of an *Ulva* biomass-based biorefinery to supply biofuels and feed to an average town in coastal India^10^,^17^. In another work, using life-cycle analysis, we have shown the advantage of *Ulva* feedstock for biofuel production in comparison with corn and cassava fresh roots in terms of land, potable water, fertilizer and herbicide usage^17^. Other groups have already demonstrated production of acetone, ethanol and butanol from *Ulva*^1^,^5^.

To convert macroalgal biomass into fuel molecules via a biochemical pathway, the first step is to degrade the cell wall material into fermentable sugars with acid or through enzymatic reactions^18^. However, energy efficient macroalgae cell deconstruction and saccharification into fermentable sugars is still a major challenge^5^,^15^,^19^,^20^. Though acid hydrolysis or pretreatment has been widely used in the degradation process of biomass^21^,^22^,^23^, the details of the process parameters and their importance in determining the process output have rarely been reported^24^,^25^. Understanding of these parameters is important as this will enable the design of energy efficient biorefineries.

To understand how various parameters could influence the cell wall deconstruction efficiency and their comparative significance, we used Taguchi Robust Experiment Design^26^ and Partial Least Square Regression. We also used Flux Balance Analysis techniques to model the potential production of ethanol, butanol and acetone from the *Ulva* biomass hydrolysates.

## Materials and Methods

### Macroalgae biomass Material

**Figure 1:**
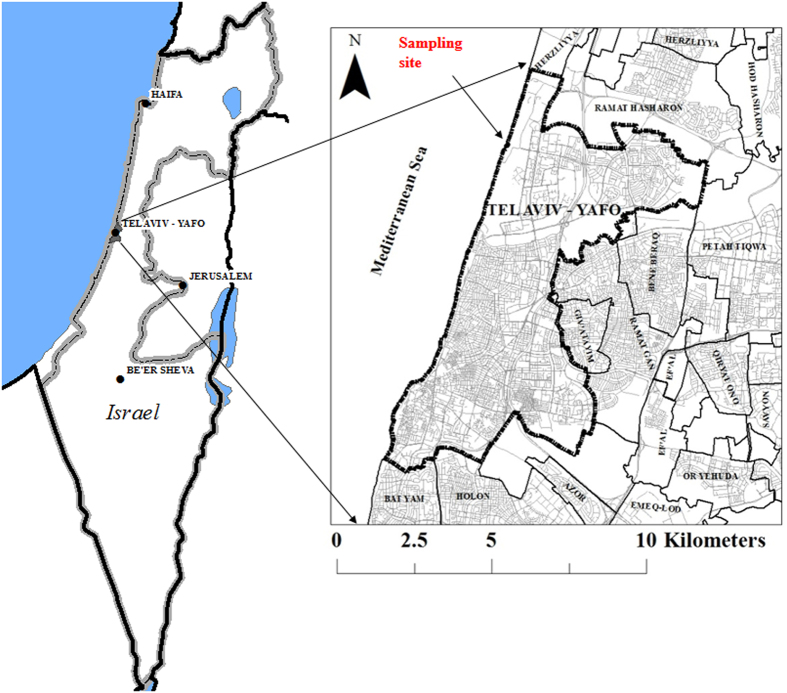
Macroalgae biomass sampling site (ArcGIS [version 10.3], (http://www.esri.com/software/arcgis)).

Macroalgae from *Ulva* genus were collected near the beach of Ramat Aviv, Israel (Fig. 1), in May 2015. The biomass dried in an oven at 40 °C until constant weight. The dried biomass was made brittle by liquid nitrogen and then grinded into powder manually in a mortar. The *Ulva* powder was sieved by 30 mesh sieve to make sure all particle sizes are smaller than 0.5 mm. All chemicals and standards were purchased from Sigma-Aldrich (Israel) if not otherwise mentioned.

### Thermochemical deconstruction

Thermochemical deconstruction was conducted in 10 mL centrifuge tubes (Nalgene™ Oak Ridge High-Speed PPCO Centrifuge Tubes (Thermo-Fisher Scientific, CA) in autoclave (Tuttnauer 2540MLV, Netherlands). For each batch, dried samples of Ulva was weighed on analytical balance (Mettler Toledo, Switzerland) Sulfuric acid (Sigma-Aldrich, Israel) was injected into the tube and the mix was vortexed to make the powder well distributed in acid. After deconstruction, the hydrolysates were neutralized by sodium hydroxide (Sigma-Aldrich, Israel). All the solid/liquid ratio, acid concentrations, hydrolysis time and temperature were according to the Taguchi design in Table S1.

### Taguchi orthogonal arrays for thermochemical deconstruction

The goal in these series of experiments was to determine the effects of thermochemical hydrolysis process parameters: temperature, treatment time, %Acid and %Solid load on the extraction of monosaccharaides from *Ulva* biomass. The possible range of process parameters and their combinations is large. Therefore, to decrease the number of experiments, but still be able to evaluate the impact of each parameter independently, we applied the Taguchi’s Robust Design method for the experimental design and analysis^26^,^27^. A key feature of the Taguchi method is to determine the parameters of the controllable process factors with the goal to minimize the impact of uncontrollable factors (noise) in industrial process. The experimental design with orthogonal arrays allows for analysis that prioritizes the comparative impact of the process parameters on the yields.

The experiments, conducted for the L16 orthogonal Taguchi array, which are needed to determine the individual effects of each of the tested parameters on the extraction yields are summarized in Table S1. The hydrolysis (1ml total volume) was conducted in 10 ml tubes (Thermo-Fisher Scientific, CA). The resulting hydrolysates were neutralized by 5 M KOH to pH 7. All experiments were done in duplicate.

In Taguchi design of experiment the best parameter setting is determined using signal-to-noise ratio (*R*). In our experiments we used “*the larger the better*” algorithm type. The ratio *R* is determined independently for each of the process outcomes (*OUT*) to be optimized. These process outcomes are: concentrations of glucose, rhamnose, xylose, glucuronic acid and total sugars, solution density and %Yield. In the current context, maximizing *R* corresponds to obtaining the maximum concentration and extraction yields of monosaccharaides. The ratio *R* of a specific process outcome *OUT* in experiment *j* was calculated by:


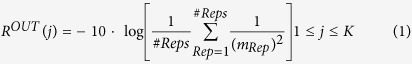


where *K* is the number of experiments (in our case *K* = 16); *#Reps* is the number of experiment repetitions (in our case *#Reps* = 2) and *m*_*Rep*_ is the measurement of the process outcome (*OUT)* in the specific repetition *Rep* of experiment *j*.

Consider a process parameter *P* (temperature, treatment time, %Acid, %Solid as appears in Table S1). Assume that P has a value of *L* in *n(P,L)* experiments (for example, temperature = 100 appears in 5 experiments: *P* = temperature, *L* = 100 and *n(P,L)* = 5). Let *J (P,L)* be the set of experiments in which process parameter *P* was applied at level *L*. Let:


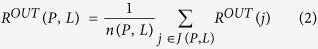


be the average ratio *R* for concrete level *L* of parameter *P*. The sensitivity (Δ) of each outcome (*OUT*) with respect to the change in a parameter *P* is calculated as:


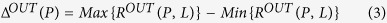


Ranking (on the scale of 1–4, where 1 is the highest) was assigned to the process parameters according to the sensitivity ranges obtained.

### Partial least squares regression

The ultimate goal of multivariate regression analysis is to create a calibration equation (or set of equations) that, when applied to data of “unknown” samples, measured in the same manner, will accurately predict the quantities of the constituents of interest. The multivariate calibration models were generated using Partial Least Squares (PLS) regression, with the goal of defining a relationship between different process parameters (*P*_*i*_) and any process output:


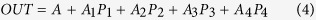


where *OUT* is the output (e.g. glucose concentration, rhamnose concentration, xylose concentration, glucoronic acid concentration, total sugars concentration, %Yield or density), A is an empirical coefficient, and *P*_*i*_ are the process parameters (temperature, treatment time, %Acid, %Solid, as appears in Table S1). For each output (*OUT*), a model consisting of four different process parameters (e.g., six models in total) was constructed. First, we investigated the influence of each *P* on each *OUT* using the whole set of physical variables. Then, we used Martens’ Uncertainty Test (MUT) to identify variables that are most important to predict different sugar concentrations, release yields, and hydrolysate properties. MUT is a significance testing method that can be implemented when cross validation PLS method is applied. It tests the stability of the regression results and the selection of significant *P*-variables (Unscrumbler, Version 9.1, Camo, Norway)^28^.

Cross-validation is the best model and, indeed, the only alternative we have when we lack sufficient samples for a separate test set^28^. Due to the limited number of samples, statistical parameters for the calibration model were calculated by leave-one-out-cross-validation (only one sample at a time is kept out of the calibration and used for prediction). The performance and relevance of PLS models were further evaluated by computing different statistics. The difference between the predicted and measured sugar contents was expressed as a root mean square error of prediction RMSECV (root mean square error of cross-validation). RMSECV is defined as the square root of the average of the squared differences between the predicted and measured values of the validation objects^28^:


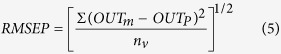


where *OUT*_*m*_ is the parameter of the sample (m), *OUT*_*P*_ is the predicted value of the sample and *n*_*v*_ is the number of samples in the calibration stage. The predictive capability of all models was compared in terms of the relative standard error for cross- validation sets (denoted as RMSECV (%)).


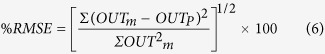


Additionally, we used the Ratio of Prediction to Deviation (RPD), which is defined as the ratio of the standard deviation of the measured values to the root mean square error of cross-validation (RMSECV) or prediction (RMSEP)^29^. An RPD value below 1.5 was taken to indicate that the model is unusable, between 1.5 and 2.0 indicated that the model has the potential to distinguish between high and low values, and an RPD value between 2.0 and 2.5 was indicative that quantitative prediction is possible. RPD values above 2.5 were considered to indicate that the predictive capability of the model is excellent.

The log10 IR was calculated to normalize the rhamnose, glucose, xylose and glucuronic acid distributions and all further quantitative analyses were made on both, the transformed and untransformed data^28^.Therefore, both, separately for each OUT_p_, were used as reference values in our PLS modelling.

### Carbohydrate composition analysis by High Performance Ion Chromatography

Dionex ICS-5000 platform (Thermo Fischer Scientific, MA, USA) was used to quantify released monosaccharaides in hydrolysate. Carbopac MA1 and its corresponding guard column were used for separation. Electrochemical detector with AgCl as reference electrode was used for detection. A trinary solvent system was used for elution as shown in Table S2. The column temperature was kept at 30 °C and the flow rate was set to 0.4 mL min^−1^. Calibration curves were produced for rhamnose, glucose, xylose and glucuronic acid on gradient to determine the concentration of corresponding substances in the hydrolysate. All uronic acid peaks were integrated and calculated accordingly using the calibration curve of glucuronic acid (UA) for estimation.

Total yield was calculate using the following Eq. 7:


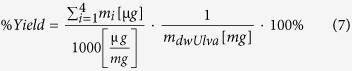


where *m*_*i*_ (*μg*) is the mass of carbohydrate *i* in the hydrolysate sample (Total), m_*dwUlva*_(mg) is the total weight of the hydrolysed biomass in each sample. The summed carbohydrates were glucose, xylose, rhamnose and glucuronic acid. The concentrations of the rest of the released monosaccharaides were negligible.

### Density determination

To determine the density of hydrolysates we used the resonating micromembranes (RMMs) method. RMMs operate in deposited liquid droplet environment^30^,^31^,^32^, characterizing the velocity response in the frequency domain^33^,^34^. Figure S1 shows a typical response spectrum of a dry RMM device of 500 μm excited near the first three fundamental modes, denoted by *(01)*, *(11)* and *(21)*. Downward frequency shifts of specific vibration modes correlate with added droplets mass^30^,^31^,^32^ as detailed below.

The resonance frequency in each mode, of indices *(mn)*, is inversely proportional to the square root of effective mass density, which contains contributions from both the solid membrane film and deposited liquid material above it^30^,^31^,^32^. The analytical relationship, corresponding to symmetrical geometry of a circular membrane, is:


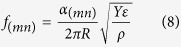


where α_(mn)_ is a geometrical factor associated Bessel function zeros along membrane nodes^30^,^31^,^32^, *Y* = 130 (GPa) is the film Young’s modulus, *ε* = 4∙10^−6^ is the pre-calibrated tensile stress, and ρ is the effective mass density including both device and deposited droplet contribution. With the known density of Silicon ρ_f_ = 2650 (kg m^−3^) and intrinsic film volume V_f_ = πR^2^h = 0.59 (pL), the deposited liquid is characterized by an average density ρ_1_ and a total droplet volume V_1_, defining the composite effective masses density:


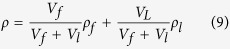


The downward frequency shift corresponding to an added mass relative to the a dry RMB is estimated using^30^:


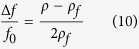


In differential measurements of frequency shifts corresponding to given modes, the pair of Eqs 9 and 10 enable us to extract both parameters ρ_1_ and V_1_ in each experiment. Here we chose to work with mode *(34)*, vibrating at f(34) = 82 KHz in the dry settings. The spectral width of each mechanical resonance is proportional both to dissipation and density. Only the average density results are used in the hydrolysates analysis below.

### Predicted ethanol yield modeling

Mathematical simulations of biomass utilization for ethanol, butanol and acetone production yields were performed according to a commonly used Flux Balance Analysis (FBA) methodology, which is a sub-class of Constraint-Based Modeling (CBM) mathematical modeling frameworks. This method analyzes internal reaction fluxes based solely on simple physical-chemical constraints, such as reaction stoichiometry and metabolic flux constraints, without requiring exact enzyme kinetic data^35^. This methodology enables the prediction of organism growth rates, as well as the prediction of minimal and maximal production rates of various metabolic compounds, based only on reaction stoichiometry and directionality. FBA-based approaches have a wide range of applications, including phenotype analysis, bioengineering and metabolic model reconstructions^36^,^37^,^38^,^39^.

In our case, we utilized FBA mathematical framework to predict production rates of ethanol, butanol and acetone from the hydrolyzed *Ulva* biomass. According to Flux Variability Analysis (FVA) approach^40^, we estimate minimal and maximal limits for the flux through the target molecule (ethanol) transporter reaction as follows. First, the maximal possible organism growth rate (*MaxGrowth*) under given media is estimated in (Eq. 11):


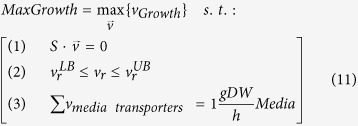


where *S* is the organism-specific stoichiometric matrix (*S*_*mr*_ corresponds to stoichiometric coefficient of metabolite *m* in the reaction *r*) and 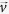 is a vector of reaction fluxes in the organism (*v*_*r*_ is a metabolic flux through reaction *r*). *v*_*Growth*_ is an artificial reaction representing organism growth rate (converting several molecules like amino acids, nucleotides and others into units of biomass) and *v*_*media transporters*_ refer to the set of transporters active for the received growth media. Constraint (1) is mass-balance constraint, enforcing the sum of fluxes for all reactions producing each metabolite to be equal to the sum of fluxes consuming it. Constraint (2) is boundary constraint, enforcing all reactions to be in their feasible range of *v*^*LB*^ and *v*^*UB*^ (which is *[-Inf;Inf]* for bidirectional and *[0;Inf]* for unidirectional reactions if no additional information is available). Constraint (3) is media constraints, limiting the media consumption to 1gDW*h^−1^.

In case of *MaxGrowth* below *ε* = *10*^*−5*^, the organism is defined as non-viable and it does not produce target molecule. Otherwise, minimal and maximal target molecule (ethanol, butanol, acetone) production rates are estimated in (Eq. 12) under the maximal growth rate constraint (a common assumption in FBA-based simulations):


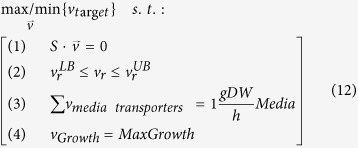


where *v*_*target*_ is the flux through the target-molecule transporter.

Specifically, to predict the biofuel molecule production rates, we utilized one-step simulation framework described in previous work^39^ with metabolic models of *S. cerevisiae*^41^ (original and modified to incorporate Xylose uptake, simulating RN1016 strain), of *E. coli*^42^ and of *C. acetobutylicum*^43^. The composition of the *Ulva* biomass was based on literature^12^,^13^,^44^ and appears in Table 1. The carbohydrate concentrations were modified for the simulations to those of rhamnose, glucose, xylose and glucoronic acid measured in experiments 1–32 (Table S10). Maximal and minimal ethanol production rates were estimated for 4 organisms: (i) *E. coli*; (ii) *S. cerevisiae WT*; (iii) *S. cerevisiae RN1016*; and (iv) *C. acetobutylicum*. For *C. acetobutylicum*, we also investigate maximal and minimal production rates of acetate and butanol.

## Results and Discussion

### *Ulva* biomass hydrolysate carbohydrate analysis

First, we quantified the released quantities of major released carbohydrates from the dried *Ulva* biomass at each experimental condition. The results are shown in Table 2.

**Figure 2:**
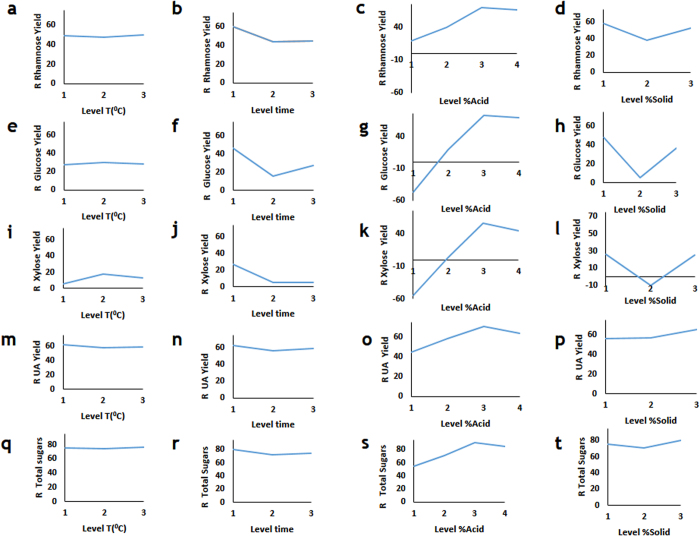
Taguchi signal-to-noise analysis (*R*) of the process parameters impact on the release of monosaccharaides from *Ulva* biomass. The effect of Temperature (**a**), Time (**b**), %Acid (**c**) and % Solid (**d**) on Rhamnose release. The effect of Temperature (**e**), Time (**f**), %Acid (**g**) and % Solid (**h**) on Glucose release. The effect of Temperature (**i**), Time (**j**), %Acid (**k**) and %Solid (**l**) on Xylose release. The effect of Temperature (**m**), Time (**n**), %Acid (**o**) and % Solid (**p**) on Glucuronic acid release. The effect of Temperature (**q**), Time (**r**), %Acid (**s**) and % Solid (**y**) on Glucuronic acid release.

**Figure 3:**
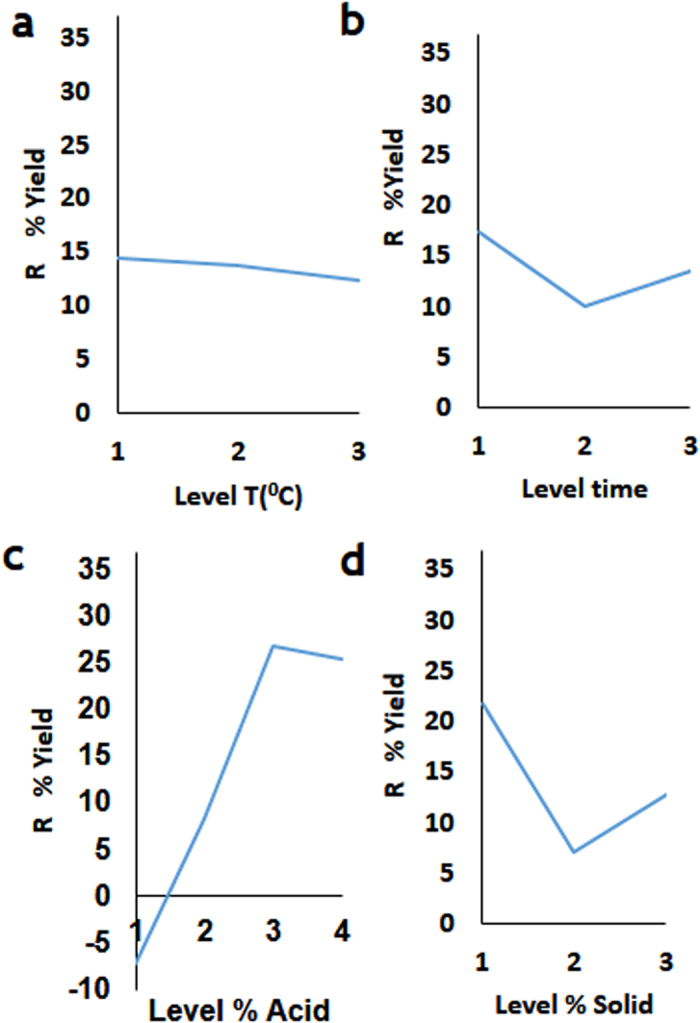
Taguchi signal-to-noise analysis (*R*) of the process parameters impact on the %*Yield* of from *Ulva* biomass. The effect of (**a**) Temperature (**b**) Time (**c**) % Acid (**d**) %Solid

We also analyzed the sensitivity of the specific and total sugar release during hydrolysis to the tested process parameters that can be controlled (Figs 2 and 3, Tables 2 and 3). Increase of temperature did not significantly affect the rhamnose, glucose and xylose yields (Fig. 2a,e,i). Shorter time increased rhamnose, glucose and xylose yields (Fig. 2b,f,j). Increasing %Acid to a certain level led to higher rhamnose, glucose and xylose yields, further increase in acid concentration did not increase the yields of these monosaccharide’s (Fig. 2c,g,k). Low and high %Solid led to the increase of the rhamnose, glucose and xylose yields (Fig. 2d,h,l).

The yields of glucoronic acid were not affect by process time and temperature (Fig. 2m,n). As with other hydrolysis products, increase of %Acid increasing %Acid to a certain level led to higher yields. Increasing %Solids increased the yield.

Process temperature and time did not affect the total concentration of released monosaccharaides (Fig. 2q.r). Increase of %Acid increasing %Acid to a curtain level led to higher yields (Fig. 2s). Increasing %Solids increased the yield (Fig. 1t).

We also found that changes in temperature did not affect the total yield (as defined in Eq. 7) of carbohydrates (Fig. 3a). Shorter of longer process times led to the increased yields (Fig. 3b). Increase in the %Acid to a certain level increased the total yield, and high %Acid led to reduction of the total yield (Fig. 3c). Low and higher %Solid load led to higher yields (Fig. 3d).

**Figure 4:**
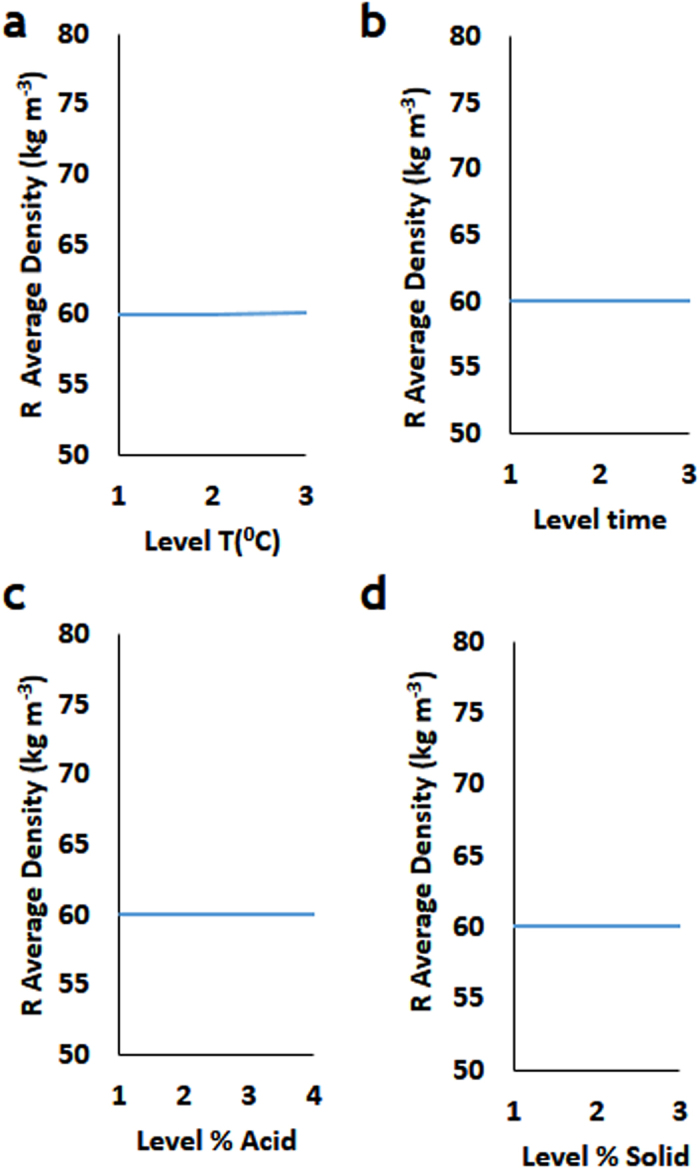
Taguchi signal-to-noise analysis (*R*) of the process parameters impact on the hydrolysate density. The effect of (**a**) Temperature (**b**) Time (**c**) % Acid (**d**) %Solid

Changes of the process parameters did not significantly affect the hydrolysate density (Fig. 4).

The average *R* values for each process factor (*P)* appear in Tables S3–S9 (see Supplementary information). The importance of the change in each of the process parameters on *Ulva* deconstruction appears in Table 4.

The optimum parameters that maximize each of the output factors (*OUT*) appear in Table 5.

### PLS analyses of macroalgae *Ulva*

As previously noted, a PLS model constructed for each chemical constituent was first implemented on the whole four physical parameters, and then only the significant parameters were kept and each model was re-assessed. The results are shown in Table 6 (significant variables highlighted by + and bold in the text).

Tables 6 and 7 present the results of PLS modelling of different sugar contents. As can be seen, acidity and solidity were found to be important variables for sugar component estimation. Highest PRD values were estimated for glucose, rhamnose and density. RPD value between 1.5 and 2.0 indicate that the model has a strong potential to distinguish between high and low values of Rhamnose and density. For Glucose, an RPD value of 2.02 indicates that a quantitative prediction is possible using acidity and solidity.

Due to the relatively small number of samples and relatively small dynamic range of reference values (e.g. released monosaccharaides concentrations), even extremely small differences between the PLS model and reference values can result in increased values of RMSE (%) (Table 8). Future modeling should be conducted on a larger data set with more reference variables.

The results from the PLS analysis further corroborate the results from Taguchi ranking analysis (Tables 4 and 5), indicating that in the tested range of parameters, %Acid and %Solid play a more important role on the hydrolysis success than the process time and temperature.

### Predicted Ethanol production

We predicted possible minimal and maximal ethanol production rates for each of 32 *Ulva* biomass hydrolysis experiments and for *Ulva* biomass media without any carbohydrates using (i) *E. coli* (Table 8); (ii) *S. cerevisiae WT* (Table 9); (iii) *S. cerevisiae RN1016* (Table 10); and (iv) *C. acetobutylicum* (Table 11). The carbohydrate content used in the model (gr kg^−1^) in Table 1 was updated based on the results from the current hydrolysis experiments (Table S10) In addition, minimal and maximal possible acetate and butanol production rates were evaluated for *C. acetobutylicum* (Table 11).

For *E.coli*, the maximum predicted production rate of ethanol is obtained at experiments 8 and 9 (19.459–20.833 gr ethanol kg^−1^ *Ulva*) (Table 8). This corresponds to the highest extraction yields of glucose with thermochemical hydrolysis. For *S. cerevisiae WT* the maximum predicted ethanol rates are 49.323–51.929 gr ethanol kg^−1^ *Ulva* DW and were also observed for simulated fermentation of hydrolysates from experiments 8 and 9 (Table 9). This also corresponds to the highest extraction yields of glucose thermochemical hydrolysis. Previous experimental studies reported on 62 gr ethanol production per kg of *Ulva pertusa* after enzymatic hydrolyses^45^,^46^.

From all simulations, the maximum production of ethanol was achieved with *Ulva* fermentation using *S. cerevisiae RN1016* (Table 10). For this organism, in simulations 1, 2, 5, 6, 11, 15 and 16 the predicted ethanol production rates were the same as for media without any monosaccharides (0–8.746 g kg^−1^) derived from hydrolysates. For other simulations we observed an increase in minimal ethanol production rates from 0 up to 54.134 g ethanol kg^−1^ *Ulva* (in exp. 8) and an increase in maximal ethanol production rate from 8.746 g ethanol kg^−1^ *Ulva* up to 57.500 g ethanol kg^−1^ *Ulva* (in exp. 8). The achieved predicted results seem reasonable, since the major carbohydrates source for ethanol is glucose, reaching maximal concentration of 93.53 g ethanol kg^−1^ *Ulva* in exp. 8. Also, these findings are supported in previous work^47^, which predicted 160 g of ethanol per kg of *Ulva*, with glucose concentration of 310 g ethanol kg^−1^ *Ulva* (keeping similar glucose-to-ethanol mass ratio of 2:1).

For *C. acetobutylicum*, in simulations with growth rates not close to zero, the non zero ABE production rate varied from 1.630 to 2.909 gr ABE kg^−1^ *Ulva* (Table 11). The ABE yield of predicted ABE fermentation of *Ulva* hydrolysate by *C. acetobutylicum* (calculated by ratio of the sum of ABE products to total sugars extracted with hydrolysis) was 0.02–0.17 range. In the reported experimental ABE, yield of *Ulva* hydrolysate fermentation by *C. acetobutylicum* was in the 0.03–0.32 range for hydrolysates with various supplements such as glucose, xylose and nutrients as in CM2 medium^1^. The ABE yield of the hydrolysate without supplements was 0.08^1^. In our simulations, the maximum predicted ABE yield is 23.955–26.830 gr ABE kg^−1^ *Ulva*. However, these results were shown for experiments 2, 5 and 15, where the growth rates of the organisms were close to 0 and these results should therefore be treated with care, as they depend on the organism survival at this specific medium. Specifically, in our simulations (Eq. 11) we first maximize organism growth rate, utilizing all available media components in favor of this task and, only then, estimate the molecule production range. This is the reason that media with more sugars does not necessarily lead to higher ABE production but rather to higher organism growth rates. On the other hand, when the amount of monosaccharaides (specifically, glucose) is very low, it acts as a limiting factor to the *C. acetobutylicum* growth, leading to near-ε (Eqs 11 and 12) growth rates (like in exp. 2, Table 2), the amount of media components not utilized by biomass-constructing reaction (*v*_*Growth*_, Eqs 11 and 12) is relatively high. Therefore, simulations allow molecule production estimation in such low growth rate scenarios. The limitation of this approach is that since ε is arbitrary, configurations with near-ε growth rates may not be viable. Moreover, if minimal molecule (i.e. ethanol) production in these scenarios is zero, ethanol production is not coupled with organism growth (i.e., the organism can grow completely without producing ethanol).

## Conclusions

In this study we investigated the impact of thermochemical hydrolysis process parameters: temperature, treatment time, %Acid and %Solid load on green macroalgae *Ulva* biomass. The experiments were designed using Taguchi orthogonal arrays approach to study the impact of the change in each of the hydrolysis parameters and the significance of each parameter on the sugars release. We found that in the studied range of values, %Acid plays the most important role and temperature plays the least important role in the monosaccharaides release. None of the factors played significant role in the density change of hydrolysates. In addition, PLS regression analysis was used to identify which variables influence different monosaccharaides concentrations. Based on our results, acidity was found to be the most important variable for all sugar types. Furthermore, we compared the possibility to produce biofuels from the *Ulva* hydrolysate using 4 different microorganisms using Flux Balance Analysis models. We showed that the highest yields of conversion, 54.134–57.500 gr ethanol kg^−1^ *Ulva* can be achieved using hydrolysate fermentation to ethanol by *S. cerevisiae* RN1016 with xylose isomerase, and when the hydrolysis conditions of 121 °C, 30min 2% Acid, 15% Solid are used. This paves the way to optimized marine algae utilization process design, and enables smart energy harvesting by thermochemical hydrolysis.

## Additional Information

**How to cite this article**: Jiang, R. *et al*. Thermochemical hydrolysis of macroalgae *Ulva* for biorefinery: Taguchi robust design method. *Sci. Rep*. **6**, 27761; doi: 10.1038/srep27761 (2016).

## Supplementary Material

Supplementary Information

## Figures and Tables

**Table 1:** Composition of *Ulva* biomass (in this case *U. lactuca)* as reported in the literature^12^,^13^,^43^ with modifications accordingly to the experimental yields.

Compound	Weight (gr kg^−1^)	Modifications in the current simulations
D-Glucose	403.4788	updated according to hydrolysis experiment (Table 2)
L-Rhamnose	195.0048	updated according to performed hydrolysis experiment (Table 2)
D-Xylose	106.028	updated according to performed hydrolysis experiment (Table 2)
D-Galactose	10.8102	removed
D-Mannose	2.9	removed
L-Arabinose	5.0642	removed
L-Aspartic acid	10.9134	
L-Threonine	5.33826	
L-Serine	5.87124	
L-Glutamic acid	10.94724	
L-Proline	3.48552	
Glycine	5.6259	
L-Alanine	7.74936	
L-Valine	7.7409	
L-Methionine	2.01348	
Cystine	1.64124	
L-Isoleucine	4.02696	
L-Leucine	6.9795	
L-Tyrosine	5.06754	
L-Phenylalanine	2.2842	
L-Histidine	1.17594	
L-Lysine	5.499	
L-Arginine	5.23674	
Myristic acid	1.8888	
Palmitic acid	46.7478	
Palmitoleic acid	5.40669	
Stearic acid	1.47169	
Oleic acid	12.53691	
Linoleic acid	1.91241	
alpha-Linolenic acid	2.5184	
Arachidic acid	0.88931	
Eicosenoic acid	1.19624	
Behenic acid	3.28179	
Docosahexaenoic acid	0.8657	
Glucuronic Acid	47.1232	updated according to experiment (Table 2)
L-Iduronic Acid	36.2304	
Other undigested particles	23.04824	updated according to experiment (Table 2)

**Table 2:** Major carbohydrates released from dried *Ulva* biomass after thermochemical hydrolysis.

*#j*	T (°C)	Time (min)	Acid (%)	Solid (%)	Rha (μg ml^−1^)	Glc (μg ml^−1^)	Xyl (μg ml^−1^)	UA (μg ml^−1^)	Total (μg)	Yield (%)	Density (kg m^−3^)
1	100	30	0	5	218.7 ± 5.2	0.2 ± 0.1	0.4 ± 0.1	238.6 ± 163.6	1236.4 ± 163.6	2.5 ± 0.3	1000.5 ± 0.6
2	100	45	0.5	15	228.7 ± 10.3	3.2 ± 0.1	5.5 ± 0.125.0	1649.9 ± 38.4	5095.5 ± 75.6	3.4 ± 0.1	1001.5 ± 0.3
3	100	60	2	25	3036.1 ± 51.3	6538.8 ± 301.5	1339.9 ± 13.6	16010.4 ± 1111.5	72697.7 ± 4020.8	29.1 ± 1.6	1000.2 ± 0.1
4	100	45	5	5	1179.1 ± 13.3	1422.4 ± 35.5	149.7 ± 1.9	1058.9 ± 149.1	10287.2 ± 571.1	20.6 ± 1.1	1004.2 ± 0.3
5	121	30	0.5	25	49.2 ± 6.7	4.3 ± 1.6	4.8 ± 0.0	942.4 ± 218.6	2702.2 ± 617.7	1.1 ± 0.2	1001.6 ± 0.2
6	121	45	0	15	3.0 ± 0.1	0.001 ± 0.0	0.0 ± 0.0	329.7 ± 277.1	898.1 ± 748.3	0.6 ± 0.5	1003.7 ± 0.1
7	121	60	5	5	1215.8 ± 93.7	1389.3 ± 89.2	82.9 ± 29.0	889.5 ± 22.8	9659.3 ± 446.2	19.3 ± 0.9	1002.8 ± 0.1
8	121	30	2	15	3739.7 ± 185.2	5196.2 ± 124.2	675.6 ± 29.0	2866.8 ± 71.6	33691.4 ± 1107.2	22.5 ± 0.7	1000.6 ± 0.2
9	134	30	2	25	6879.6 ± 43.2	8137.7 ± 36.6	1054.2 ± 4.8	3887.0 ± 21.9	53888.2 ± 287.5	21.6 ± 0.1	1001.5 ± 0.1
10	134	45	5	25	5218.8 ± 284.5	7606.6 ± 253.7	300.4 ± 3.2	2829.6 ± 77.7	43079.3 ± 1671.7	17.2 ± 0.7	1013.1 ± 0.4
11	134	60	0	15	3.2 ± 0.4	0.01 ± 0.001	0.01 ± 0.001	211.7 ± 101.7	580.3 ± 275.7	0.4 ± 0.2	1008.2 ± 1.1
12	134	60	0.5	5	976.1 ± 87	1172.3 ± 138.3	128.6 ± 11.7	744.4 ± 32.4	8157.7 ± 727.7	16.3 ± 1.5	1033.9 ± 1.8
13	134	30	5	15	3510.8 ± 445.1	4691.1 ± 554.9	209.4 ± 33.7	2120.7 ± 222.1	28436.4 ± 3390.7	19.0 ± 2.3	1008.7 ± 0.9
14	121	45	2	5	1109.7 ± 12.3	1255.2 ± 22.0	142.2 ± 17.3	836.3 ± 178.0	9027.0 ± 553.6	18.1 ± 1.1	1014.8 ± 0.6
15	100	60	0.5	15	10.8 ± 0.7	0.6 ± 0.1	0.4 ± 0.6	456.0 ± 91.9	1263.2 ± 248.4	0.8 ± 0.2	1004.3 ± 2.3
16	134	45	0	25	2.9 ± 0.1	0.01 ± 0.001	0.01 ± 0.001	126.0 ± 17.8	348.2 ± 48.0	0.1 ± 0.0	1008.0 ± 0.5

**Table 3:** Taguchi *R* ratio for “larger the better” case.

*#j*	T (°C)	Time (min)	Acid (%)	Solid (%)	R Rha	R Glc	R Xyl	R UA	R Total	R %Yield
1	100	30	0	5	46.79	−7.64	−36.99	47.21	61.73	7.75
2	100	45	0.5	15	47.17	10.03	14.80	64.35	74.14	10.62
3	100	60	2	25	69.64	76.30	62.54	84.06	97.21	29.25
4	100	45	5	5	61.43	63.06	43.45	60.37	80.23	26.25
5	121	30	0.5	25	33.73	11.82	12.67	59.13	68.29	0.33
6	121	45	0	15	9.39	−60.00	−60.00	45.27	54.07	−9.45
7	121	60	5	5	61.66	62.83	38.35	58.98	79.69	25.71
8	121	30	2	15	71.44	74.31	56.58	69.14	90.54	27.02
9	134	30	2	25	76.75	78.21	60.46	71.79	94.63	26.67
10	134	45	5	25	74.33	77.62	49.55	69.03	92.68	24.72
11	134	60	0	15	9.99	−60.00	−60.00	44.98	53.77	−9.75
12	134	60	0.5	5	59.74	61.29	42.13	57.42	78.18	24.20
13	134	30	5	15	70.80	73.33	46.25	66.46	88.98	25.46
14	121	45	2	5	60.90	61.97	42.96	58.15	79.09	25.11
15	100	60	0.5	15	20.64	−4.44	−56.99	52.91	61.78	−1.75
16	134	45	0	25	9.38	−60.00	−60.00	41.88	50.71	−17.25

**Table 4:** The ranking of the importance of process parameters on carbohydrates extraction yields (change in the parameter ranked 1 has the largest effect on the extraction yield and change in parameter ranked 4 has the lowest effect on the extraction yield).

	T (°C)	Time	%Acid	%Solid
Rhamnose	4	3	1	2
Glucose	4	3	1	2
Xylose	4	3	1	2
Glucuronic acid	4	3	1	2
Total Sugars	4	3	1	2
%Yield	4	3	1	2

**Table 5:** Optimum process parameters to maximize the hydrolysis outputs.

	T (°C)	Time	%Acid	%Solid
Rhamnose	134	30	2	5
Glucose	121	30	2	5
Xylose	121	30	2	5
Glucuronic acid	100	30	2	25
Total Sugars	134	30	2	25
%Yield	100	30	2	5

**Table 6:** Variables that are important with respect to high process outcome yield, as identified by PLS regression using Martens Uncertainty test.

*OUT*	Time	%Acid	%Solid	T (°C)	Model description
Rhamnose	+	+	+	+	Rha = 844+0.28*Temp−0.28*time+0.43*%Acid+0.32*%Solid
Glucose		+	+		Glu = 982+0.06*Temp−0.09*time+0.58*%Acid+0.50*%Solid
Xylose	+	+		+	Glu = 982+0.06*Temp−0.09*time+0.58*%Acid+0.50*%Solid
Glucouronic acid		+	+		UA = 597+0.21*Temp−0.37*time+0.35*%Acid+0.46*%Solid
Total Sugars		+	+		Total = 10064+0.06*Temp−0.1*time+0.41*%Acid+0.49*%Solid
Yield%		+			Yield = 6.44+0.04*Temp−0.004*time+0.7*%Acid−0.60*%Solid
Density	+		+	+	Density = 440+0.66*Temp+0.35*time−0.10*%Acid−0.39*%Solid

“+” shows the process factors that were identified as important to increase the yields of the specific process outcome.

**Table 7:** Statistical parameters obtained for the cross-validation.

OUT	RMSEP	RMSE(%)	R	RPD
Rhamnose	1380	12.78	0.76	1.90
Glucose	1886	4.66	0.77	2.02
Xylose	333	68.5	0.45	1.23
Glucouronic acid	2726	13.9	0.68	1.40
Total Sugars	16337	13.8	0.56	1.35
%Yield	7.25	45.2	0.67	1.40
Density	5.30	2.1	0.76	1.7

**Table 8:** Predicted ethanol yields using *Ulva* hydrolysate fermentation by *E.coli*, based on BioLego fermentation simulation.

*#j*	*E.coli* growth rate (h^−1^)	Min Ethanol (g Kg^−1^)	Max Ethanol (gr Kg^−1^)
1	0.003	0.000	0.000
2	0.005	0.000	0.000
3	0.051	13.094	14.294
4	0.034	13.757	15.955
5	0.002	0.000	0.000
6	0.001	0.000	0.000
7	0.032	12.531	14.879
8	0.038	18.883	20.833
9	0.036	17.358	19.459
10	0.029	14.319	16.798
11	0.000	0.000	0.000
12	0.027	10.605	13.151
13	0.031	14.819	17.158
14	0.030	11.681	14.119
15	0.001	0.000	0.000
16	0.000	0.000	0.000
Hydrolysate free medium	0.000	0.000	0.000

**Table 9:** Predicted ethanol yields using *Ulva* hydrolysates fermentation by *S. cerevisiae* WT, based on BioLego fermentation simulation.

*#j*	*S. cerevisiae*WT growth rate (h^−1^)	Min Ethanol (g Kg^−1^)	Max Ethanol (gr Kg^−1^)
1	0.000	0.000	8.753
2	0.000	0.000	8.774
3	0.011	33.205	41.349
4	0.012	36.149	44.209
5	0.000	0.000	8.769
6	0.000	0.000	8.746
7	0.012	35.297	43.381
8	0.014	44.107	51.929
9	0.014	41.421	49.323
10	0.013	38.693	46.674
11	0.000	0.000	8.746
12	0.010	29.724	37.974
13	0.013	39.780	47.731
14	0.011	31.852	40.038
15	0.000	0.000	8.751
16	0.000	0.000	8.746
Hydrolysate free medium	0.000	0.000	8.746

**Table 10:** Predicted ethanol yields using *Ulva* hydrolysates fermentation by *S. cerevisiae RN1016 (+Xylose isomerase)*, based on BioLego fermentation simulation.

*#j*	*S. cerevisiae* RN1016 (+ Xylose isomerase) growth rate (h^−1^)	Min Ethanol (g Kg^−1^)	Max Ethanol (gr Kg^−1^)
1	0.000	0.000	8.762
2	0.000	0.000	8.820
3	0.013	44.609	47.890
4	0.013	43.441	47.741
5	0.000	0.000	8.792
6	0.000	0.000	8.746
7	0.012	38.909	45.448
8	0.016	54.134	57.500
9	0.015	51.063	54.450
10	0.013	40.960	48.174
11	0.000	0.000	8.746
12	0.011	35.896	41.096
13	0.014	42.621	49.473
14	0.012	38.753	43.428
15	0.000	0.000	8.755
16	0.000	0.000	8.746
Hydrolysate free medium	0.000	0.000	8.746

**Table 11:** Predicted ethanol, acetone and butanol yields using *Ulva* hydrolysates fermentation by *C. acetobutylicum* based on BioLego fermentation simulation.

*#j*	*C. acetobutylicum*Growth rate (h^−1^)	Min Ethanol (g Kg^−1^)	Max Ethanol (gr Kg^−1^)	Min Acetone (g Kg^−1^)	Max Acetone (g Kg^−1^)	Min Butanol (g Kg^−1^)	Max Butanol (g Kg^−1^)
1	0.000	0.000	1.719	0.000	2.176	0.000	8.281
2	0.000	0.000	3.368	0.000	4.247	0.000	16.340
3	0.079	1.348	1.348	0.282	0.282	0.000	0.000
4	0.085	1.675	1.675	0.299	0.299	0.000	0.000
5	0.000	0.000	6.073	0.000	4.302	0.000	16.455
6	0.000	0.000	0.000	0.000	0.000	0.000	0.000
7	0.083	1.581	1.581	0.296	0.296	0.000	0.000
8	0.099	2.557	2.557	0.352	0.352	0.000	0.000
9	0.094	2.260	2.260	0.334	0.334	0.000	0.000
10	0.089	1.956	1.956	0.317	0.317	0.000	0.000
11	0.000	0.000	0.000	0.000	0.000	0.000	0.000
12	0.073	0.940	0.940	0.137	0.549	0.000	0.578
13	0.091	2.078	2.078	0.325	0.325	0.000	0.000
14	0.077	1.198	1.198	0.244	0.349	0.000	0.067
15	0.000	0.000	3.433	0.000	4.340	0.000	16.548
16	0.000	0.000	0.000	0.000	0.000	0.000	0.000
Hydrolysate free medium	0.000	0.000	0.000	0.000	0.000	0.000	0.000
