# Supplementary material for: Thermochemical hydrolysis of macroalgae Ulva for biorefinery: Taguchi robust design method
Source: Sci Rep. 2016 Jun 13;6:27761. doi: 10.1038/srep27761 (PMC4904202; doi:10.1038/srep27761)

**Supplementary Information.**

**Thermochemical hydrolysis of macroalgae *Ulva* for biorefinery: Taguchi robust design method.**

Rui Jiang^1^, Yoav Linzon^2^, Edward Vitkin^3^, Zohar Yakhini^3,4^, Alexandra Chudnovsky^5^, and Alexander Golberg^1*^

^1^The Porter School of Environmental Studies, Tel Aviv University, Tel Aviv, Israel.

^2^Department of Mechanical Engineering, Tel Aviv University, Tel Aviv, Israel.

^3^Department of Computer Science, Technion – Israel Institute of Technology, Haifa, Israel.

^4^Agilent Laboratories, Tel Aviv, Israel.

^5^Department of Geography and Human Environment, Enviro-Digital Lab, Tel Aviv University, Israel.

*Correspondences should be addressed to [agolberg@tauex.tau.ac.il](mailto:agolberg@tauex.tau.ac.il)

**Table S1.** Taguchi L16 orthogonal array for determination of optimum process parameters (*P*) for thermochemical deconstruction of macroalgae *Ulva* biomass.

| ***#j*** | **T(℃)** | **Time(min)** | **%Acid** | **%Solid** |
| --- | --- | --- | --- | --- |
| 1 | 100 | 30 | 0 | 5 |
| 2 | 100 | 45 | 0.5 | 15 |
| 3 | 100 | 60 | 2 | 25 |
| 4 | 100 | 45 | 5 | 5 |
| 5 | 121 | 30 | 0.5 | 25 |
| 6 | 121 | 45 | 0 | 15 |
| 7 | 121 | 60 | 5 | 5 |
| 8 | 121 | 30 | 2 | 15 |
| 9 | 134 | 30 | 2 | 25 |
| 10 | 134 | 45 | 5 | 25 |
| 11 | 134 | 60 | 0 | 15 |
| 12 | 134 | 60 | 0.5 | 5 |
| 13 | 134 | 30 | 5 | 15 |
| 14 | 121 | 45 | 2 | 5 |
| 15 | 100 | 60 | 0.5 | 15 |
| 16 | 134 | 45 | 0 | 25 |

**Table S2.** Ion Chromatography protocol for *Ulva* hydrolysates.

| **Time (min)** | **A (water)** | **B (480 mM NaOH)** | **C (1M NaAcO)** |
| --- | --- | --- | --- |
| 0 | 94% | 6% | 0% |
| 10 | 52 | 8 | 40 |
| 12 | 22 | 8 | 70 |
| 18 | 20 | 80 | 0 |
| 20 | 94 | 6 | 0 |

**Table S3. Taguchi *R* analysis for Rhamnose**

| **Level** | T(℃) | Time(min) | %Acid | %Solid |
| --- | --- | --- | --- | --- |
| 1 | 49.14 | 59.90 | 18.89 | 58.10 |
| 2 | 47.42 | 43.77 | 40.32 | 38.24 |
| 3 | 50.17 | 44.33 | 69.68 | 52.77 |
| 4 |  |  | 67.06 |  |
| Δ | 2.74 | 16.14 | 50.80 | 19.87 |
| **Rank** | 4 | 3 | 1 | 2 |

**Table S4.** Taguchi *R* analysis for Glucose

| **Level** | T(℃) | Time(min) | %Acid | %Solid |
| --- | --- | --- | --- | --- |
| 1 | 27.46 | 46.01 | -46.91 | 48.30 |
| 2 | 30.19 | 15.45 | 19.68 | 5.54 |
| 3 | 28.41 | 27.20 | 72.70 | 36.79 |
| 4 |  |  | 69.21 |  |
| Δ | 2.73 | 30.56 | 119.61 | 42.76 |
| **Rank** | 4 | 3 | 1 | 2 |

**Table S5.** Taguchi *R* analysis for Xylose

| **Level** | T(℃) | Time(min) | %Acid | %Solid |
| --- | --- | --- | --- | --- |
| 1 | 5.36 | 27.79 | -54.25 | 25.98 |
| 2 | 18.11 | 5.13 | 3.15 | -9.89 |
| 3 | 13.07 | 5.21 | 55.64 | 25.04 |
| 4 |  |  | 44.40 |  |
| Δ | 12.75 | 22.67 | 109.88 | 35.87 |
| **Rank** | 4 | 3 | 1 | 2 |

**Table S6. Taguchi *R* analysis for Glucuronic acid.**

| **Level** | T(℃) | Time(min) | %Acid | %Solid |
| --- | --- | --- | --- | --- |
| 1 | 61.78 | 62.75 | 44.83 | 56.43 |
| 2 | 58.13 | 56.51 | 58.45 | 57.18 |
| 3 | 58.59 | 59.67 | 70.79 | 65.18 |
| 4 |  |  | 63.71 |  |
| Δ | 3.64 | 6.24 | 25.95 | 8.75 |
| **Rank** | 4 | 3 | 1 | 2 |

**Table S7.** Taguchi *R* analysis for Total sugars.

| **Level** | T(℃) | Time(min) | %Acid | %Solid |
| --- | --- | --- | --- | --- |
| 1 | 75.02 | 80.84 | 55.07 | 75.78 |
| 2 | 74.34 | 71.82 | 70.60 | 70.55 |
| 3 | 76.49 | 74.12 | 90.37 | 80.70 |
| 4 |  |  | 85.39 |  |
| Δ | 2.16 | 9.02 | 35.30 | 10.16 |
| **Rank** | 4 | 3 | 1 | 2 |

**Table S8.** Taguchi *R* analysis for hydrolysis Yield.

| **Level** | T(℃) | Time(min) | %Acid | %Solid |
| --- | --- | --- | --- | --- |
| 1 | 14.43 | 17.45 | -7.17 | 21.80 |
| 2 | 13.74 | 10.00 | 8.35 | 7.03 |
| 3 | 12.34 | 13.53 | 27.01 | 12.75 |
| 4 |  |  | 25.53 |  |
| Δ | 2.08 | 7.45 | 34.19 | 14.78 |
| **Rank** | 4 | 3 | 1 | 2 |

**Table S9.** Taguchi *R* analysis for average Density.

| **Level** | T(℃) | Time(min) | %Acid | %Solid |
| --- | --- | --- | --- | --- |
| 1 | 60.019 | 60.022 | 60.044 | 60.096 |
| 2 | 60.041 | 60.065 | 60.088 | 60.039 |
| 3 | 60.105 | 60.085 | 60.037 | 60.042 |
| 4 |  |  | 60.062 |  |
| Δ | 0.086 | 0.062 | 0.051 | 0.057 |
| **Rank** | 1 | 2 | 4 | 3 |

**Table S10.** Updates in composition of *U. lactuca* based on the hydrolysis experiments.

| **j** | **Rha**  **(gr kg^-1^)** |  | **Glc**  **(gr kg^-1^)** | **Xyl**  **(gr kg^-1^)** | **UA**  **(gr kg^-1^)** | **Total Sugars (gr kg^-1^)** | **Undigested particles**  **(gr kg^-1^)** |
| --- | --- | --- | --- | --- | --- | --- | --- |
| 1-1 | 11.61 |  | 0.00 | 0.00 | 10.80 | 22.41 | 771.04 |
| 1-2 | 12.01 |  | 0.02 | 0.04 | 14.97 | 27.04 | 766.42 |
| 2-1 | 4.25 |  | 0.06 | 0.10 | 29.21 | 33.61 | 759.84 |
| 2-2 | 3.98 |  | 0.06 | 0.10 | 30.19 | 34.33 | 759.13 |
| 3-1 | 33.18 |  | 72.92 | 14.66 | 181.40 | 302.16 | 491.29 |
| 3-2 | 32.40 |  | 68.32 | 14.28 | 164.42 | 279.42 | 514.04 |
| 4-1 | 63.17 |  | 75.45 | 7.56 | 51.48 | 197.67 | 595.79 |
| 4-2 | 64.18 |  | 78.17 | 8.60 | 62.87 | 213.82 | 579.64 |
| 5-1 | 0.58 |  | 0.06 | 0.07 | 11.85 | 12.56 | 780.90 |
| 5-2 | 0.48 |  | 0.03 | 0.04 | 8.51 | 9.06 | 784.40 |
| 6-1 | 0.05 |  | 0.00 | 0.00 | 2.41 | 2.46 | 791.00 |
| 6-2 | 0.05 |  | 0.00 | 0.00 | 9.46 | 9.52 | 783.94 |
| 7-1 | 69.23 |  | 78.43 | 4.67 | 47.16 | 199.50 | 593.96 |
| 7-2 | 62.08 |  | 71.61 | 4.28 | 48.91 | 186.88 | 606.58 |
| 8-1 | 64.96 |  | 91.95 | 11.79 | 50.69 | 219.39 | 574.07 |
| 8-2 | 69.67 |  | 95.11 | 12.53 | 52.51 | 229.83 | 563.63 |
| 9-1 | 74.63 |  | 88.17 | 11.42 | 42.15 | 216.37 | 577.09 |
| 9-2 | 73.97 |  | 87.61 | 11.35 | 41.81 | 214.74 | 578.72 |
| 10-1 | 58.54 |  | 84.09 | 3.27 | 31.15 | 177.05 | 616.41 |
| 10-2 | 54.19 |  | 80.21 | 3.22 | 29.97 | 167.59 | 625.87 |
| 11-1 | 0.05 |  | 0.00 | 0.00 | 2.52 | 2.57 | 790.89 |
| 11-2 | 0.06 |  | 0.00 | 0.00 | 5.11 | 5.17 | 788.29 |
| 12-1 | 49.38 |  | 58.02 | 6.50 | 38.96 | 152.86 | 640.59 |
| 12-2 | 56.03 |  | 68.59 | 7.39 | 41.44 | 173.45 | 620.01 |
| 13-1 | 57.53 |  | 77.38 | 3.34 | 35.35 | 173.59 | 619.87 |
| 13-2 | 68.86 |  | 91.50 | 4.20 | 41.00 | 205.56 | 587.90 |
| 14-1 | 60.39 |  | 66.94 | 7.02 | 38.36 | 172.71 | 620.75 |
| 14-2 | 59.46 |  | 68.62 | 8.34 | 51.96 | 188.37 | 605.09 |
| 15-1 | 0.19 |  | 0.01 | 0.01 | 7.04 | 7.25 | 786.21 |
| 15-2 | 0.20 |  | 0.01 | 0.00 | 9.38 | 9.59 | 783.87 |
| 16-1 | 0.03 |  | 0.00 | 0.00 | 1.50 | 1.53 | 791.93 |
| 16-2 | 0.03 |  | 0.00 | 0.00 | 1.23 | 1.26 | 792.20 |
| empty media | 0.00 |  | 0.00 | 0.00 | 0.00 | 0.00 | 793.46 |

**Figure S1. Microelectromechanical measurement of hydrolysate density. a.** Resonance response curve of the dry RMM under electrostatic actuation in the first three mechanical flexural modes, f(01)=11.4 KHz, f(11)=14.1 KHz, f(21)=16.95 KHz. **b.** Corresponding image of the RMM facet. The membrane in use is composed of poly-crystalline Silicon (poly-si) of radius R=250 mm and thickness h=3 mm.


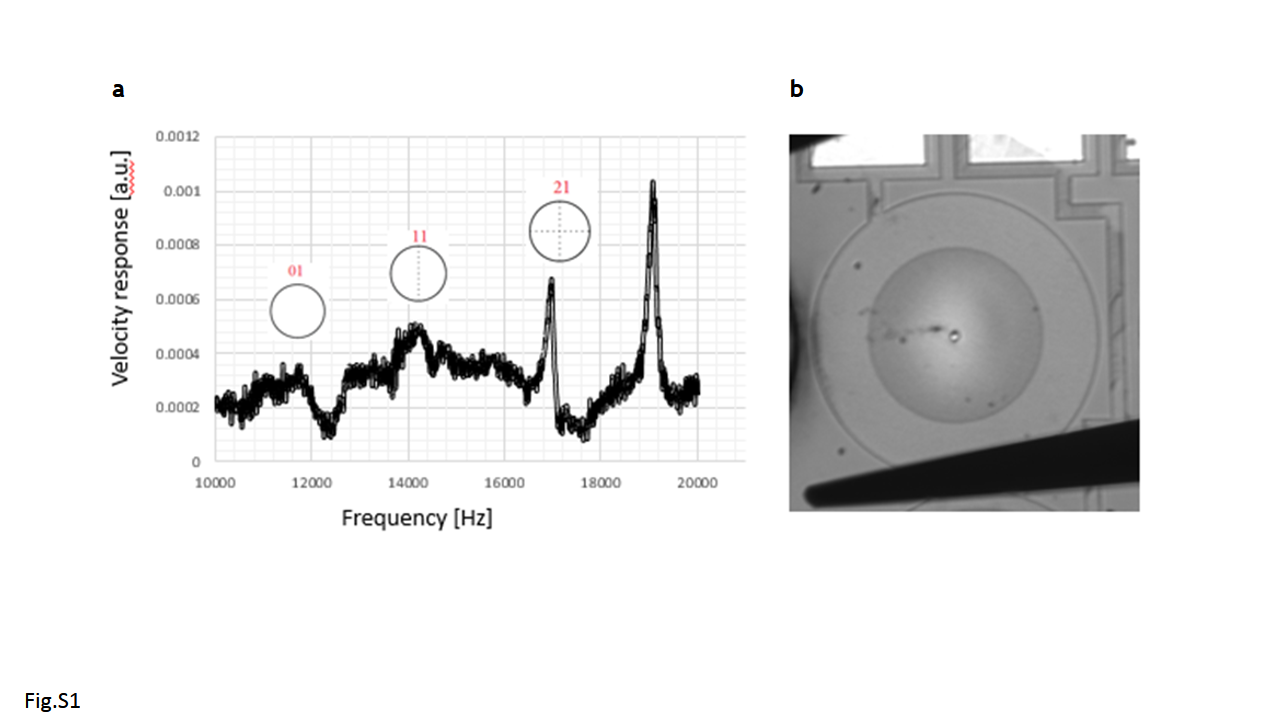

Supplement: Supplementary Information [file srep27761-s1.doc]
